# Reproducibility of free-breathing multi-slice native myocardial T_1_ mapping using the slice-interleaved T_1_ (STONE) sequence

**DOI:** 10.1186/1532-429X-17-S1-W29

**Published:** 2015-02-03

**Authors:** Jihye Jang, Sébastien Roujol, Sebastian Weingärtner, Tamer A Basha, Sophie Berg, Warren J Manning, Reza Nezafat

**Affiliations:** 1 Department of Medicine, Beth Israel Deaconess Medical Center and Harvard Medical School, Boston, MA, USA; 2 Computer Aided Medical Procedures, Technische Universität München, Munich, Germany; 3 Department of Radiology, Beth Israel Deaconess Medical Center and Harvard Medical School, Boston, MA, USA

## Background

Quantitative myocardial T_1_ mapping is a promising technique for assessment of interstitial diffuse fibrosis. Recently, a novel T_1_ mapping sequence for free-breathing, multi-slice, myocardial T_1_ mapping using the slice-interleaved T_1_ (STONE) has been developed [1], which was shown to provide superior accuracy compared to MOLLI [2]. However, in-vivo reproducibility and precision of this sequence was not studied. In this study, we sought to investigate the reproducibility and precision of the STONE sequence for in-vivo native myocardial T_1_ measurement.

## Methods

Nine healthy adult subjects (37±22y, 4 m) were scanned on a 1.5 T Philips scanner using the STONE T_1_ mapping sequence. The STONE sequence enables sampling of the undisturbed T_1_ recovery curve by selectively exciting each slice once after a single nonselective inversion pulse. The STONE sequence was implemented using a b-SSFP imaging readout and the following parameters: TR/TE=2.8/1.41ms, flip angle=70˚, FOV=280×272 mm^2^, voxel size=2×2 mm^2^, slice thickness=8 mm, 5 slices, slice gap=8mm, number of phase-encoding lines=43, linear ordering, 10 linear ramp-up pulses, SENSE factor=2.5, half Fourier=0.75. To compensate for respiratory motion, prospective slice tracking was combined with retrospective in-plane image registration [3]. The STONE sequence was compared to a single slice breath-hold MOLLI sequence which was acquired with a 5-(3)-3 scheme and similar imaging parameters. The single slice of the MOLLI corresponded to the middle slice of the STONE, which represented the mid left ventricle. Both sequences were acquired 5 times repeatedly for each subject. In-vivo measurement, precision (i.e. spatial variability) and reproducibility of T_1_ values were evaluated based on a 16 myocardial segment model for STONE and a 6 myocardial segment model for MOLLI. Precision was defined as the standard deviation of T_1_ values over each segment. Reproducibility was defined as the standard deviation of the T_1_ values over the 5 repeated scans within each segment. A paired t-test was performed on the measures of the mid left ventricle slice of STONE and MOLLI to assess for statistical significant differences between the two sequences.

## Results

**Figure 1:**
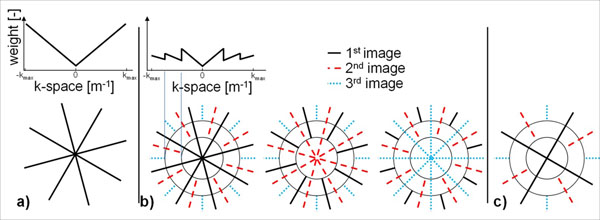
Example of in-vivo T_1_ maps of five repeated scans with the STONE and MOLLI sequences in one subject. The three mid-ventricular slices are displayed for the STONE sequence. The quality of T_1_ maps appears homogeneous and reproducible over five repetitions.

**Figure 2:**
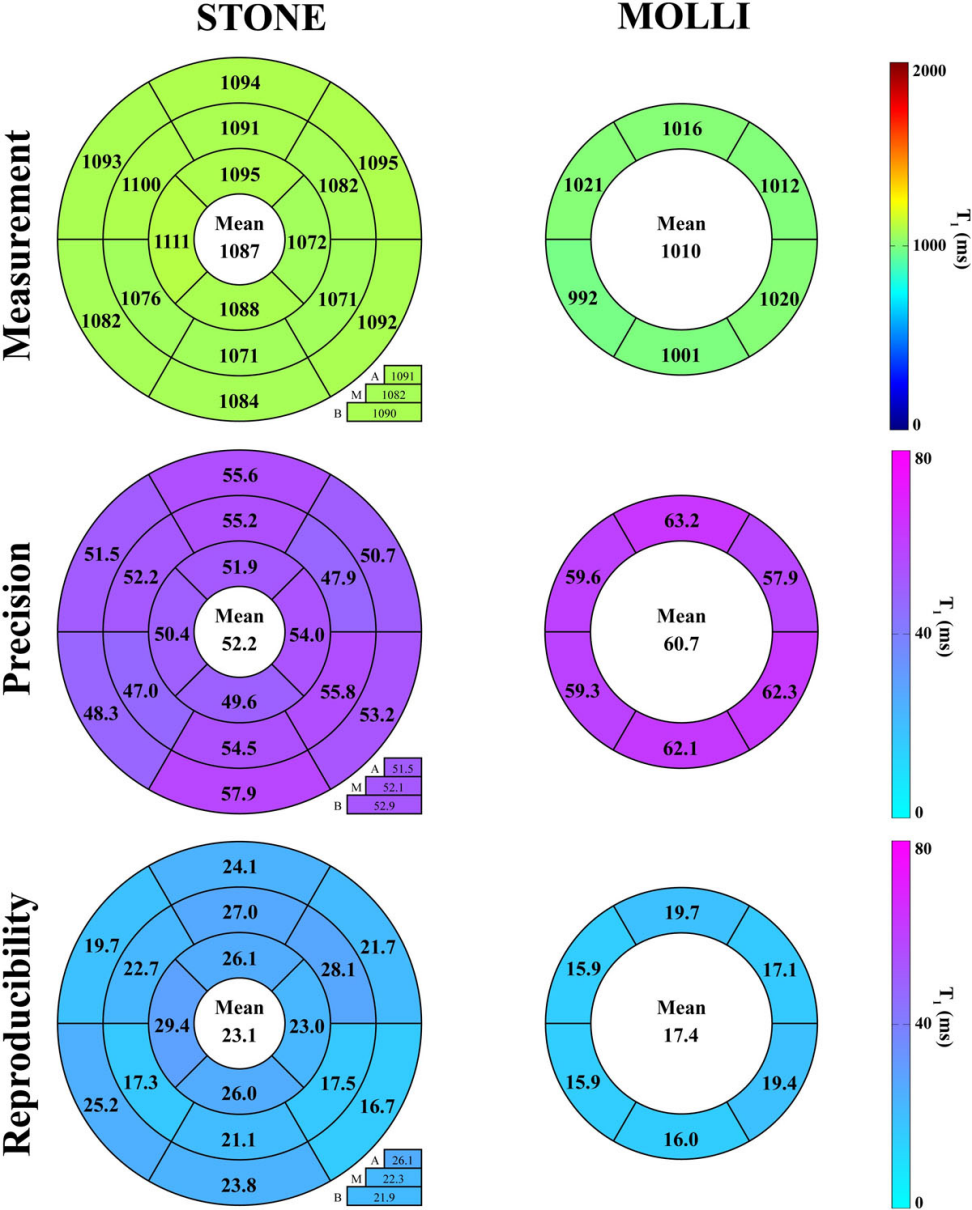
In-vivo characterization of native T_1_ times obtained with the STONE sequence and the MOLLI sequence in terms of measurement, precision and reproducibility. A 16-segment model based analysis was performed using the three mid-ventricular slices of the STONE sequence, and is compared with a 6-segment model based analysis of the MOLLI sequence using a single slice which corresponds to the middle slice of the STONE sequence. The STONE sequence yields higher accuracy (p<0.001), higher precision (p=0.001), and similar reproducibility (p=0.18) compared to MOLLI.

Figure 1 shows an example of T_1_ maps obtained in one subject. Homogenous T_1_ signals were obtained over all myocardial segments, slices, and repetitions. The STONE sequence showed higher T_1_ values (1087±35ms vs. 1010±36ms, p<0.001), higher precision (52±11ms vs. 61±16ms, p=0.001), and similar reproducibility (23±13ms vs. 17±11ms, p=0.18) than MOLLI (Figure 2).

## Conclusions

The STONE sequence yields higher T_1_ times, higher precision and similar reproducibility than MOLLI for in-vivo native T_1_ mapping.
